# Effects of GGT and C-S Lyase on the Generation of Endogenous Formaldehyde in *Lentinula edodes* at Different Growth Stages

**DOI:** 10.3390/molecules24234203

**Published:** 2019-11-20

**Authors:** Xiaoyu Lei, Shuangshuang Gao, Xi Feng, Zhicheng Huang, Yinbing Bian, Wen Huang, Ying Liu

**Affiliations:** 1 College of Food Science and Technology, Huazhong Agricultural University, Wuhan 430070, China; xiaoyulei1988@126.com (X.L.); 13720161459@163.com (S.G.); HuangZhiCheng1210@163.com (Z.H.); 2 Department of Nutrition, Food Science and Packaging, California State University, San Jose, CA 95192, USA; xi.feng@sjsu.edu; 3 Institute of Applied Mycology, Huazhong Agricultural University, Wuhan 430070, China; bianyb.123@163.com

**Keywords:** *Lentinula edodes*, endogenous formaldehyde, GGT, C-S lyase, expression levels

## Abstract

Endogenous formaldehyde is generated as a normal metabolite via bio-catalysis of γ-glutamyl transpeptidase (GGT) and L-cysteine sulfoxide lyase (C-S lyase) during the growth and development of *Lentinula edodes*. In this study, we investigated the mRNA and protein expression levels, the activities of GGT and C-S lyase, and the endogenous formaldehyde content in *L. edodes* at different growth stages. With the growth of *L. edodes*, a decrease was found in the mRNA and protein expression levels of GGT, while an increase was observed in the mRNA and protein expression levels of C-S lyase as well as the activities of GGT and C-S lyase. Our results revealed for the first time a positive relationship of formaldehyde content with the expression levels of *Csl* (encoding Lecsl) and Lecsl (C-S lyase protein of *Lentinula edodes*) as well as the enzyme activities of C-S lyase and GGT during the growth of *L. edodes*. This research provided a molecular basis for understanding and controlling the endogenous formaldehyde formation in *Lentinula edodes* in the process of growth.

## 1. Introduction

**Figure 1:**
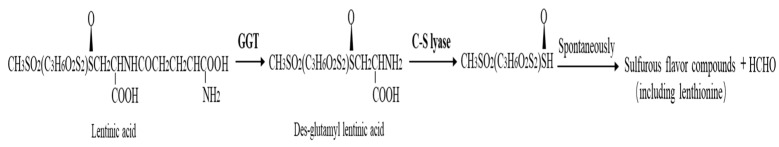
Proposed pathway for the generation of sulfurous flavor compounds and endogenous formaldehyde in *Lentinula edodes*.

*Lentinula edodes* (shiitake mushroom) is the second-most popular edible mushrooms in the world (the No. 1 is *Agaricus bisporus*), due to its high nutritional and medicinal values as well as the unique flavor [1,2,3,4]. Lenthionine (1,2,3,5,6-pentathiepane), the unique aroma of *L. edodes* [5,6], is derived from lentinic acid in a two-step enzymatic reaction [7,8]. In the reaction, the lentinic acid is catalyzed by γ-glutamyl transpeptidase (GGT) and L-cysteine sulfoxide lyase (C-S lyase) to generate the unique flavor compounds, including lenthionine [8,9,10]. Nevertheless, formaldehyde is also produced in this metabolic pathway (Figure 1).

Formaldehyde, a mutagen, can be found in the air, natural and processed foods, especially in frozen food and dry foods, and is classified as a human carcinogen by the International Agency for Research on Cancer (IARC) of World Health Organization [11]. The maximum daily dose reference for formaldehyde is defined as about 0.2 mg/kg body weight per day by the US Environmental Protection Agency. Even small doses of formaldehyde can cause various symptoms of physical discomfort [12]. However, the formaldehyde contents are 1–20 mg/kg in various fruits, vegetables, meat and fish products [13]. In recent years, the amount of formaldehyde in *L. edodes* has raised the public concerns about food safety. For instance, high levels of formaldehyde (100–300 mg/kg) have been detected in shiitake mushroom samples produced in UK and Chinese [14]. Japanese researchers have reported that formaldehyde is generated in the growth process of *L. edodes* as a normal metabolite to form its unique flavor [15].

Meanwhile, γ-glutamyl transpeptidase (GGT; EC 2.3.2.2) is an enzyme that catalyzes the transfer of the γ-glutamyl group of glutathione and related γ-glutamyl amides to water (hydrolysis) or to amino acids and peptides (transpeptidation) [16]. Cysteine sulfoxide lyase (EC 4.4.1.4) is a pyridoxal-5-phosphate (PLP) dependent enzyme and assigned to the class I family of PLP dependent enzymes [17]. In our previous work, the two enzymes were purified and characterized to play a significant biochemical role in the generation of endogenous formaldehyde in *L. edodes* [18]. Additionally, the gene of *Csl* encoding Lecsl (*L. edodes* C-S lyase) was cloned [19].

However, little is known about the relationship of GGT and C-S lyase with the production of formaldehyde in *L. edodes*. Thus, the aims of the present work were to determine the mRNA and protein expression levels and the activities of GGT and C-S lyase at different growth stages of shiitake mushrooms and to explore their correlations with endogenous formaldehyde production in *L. edodes*. This research could provide a molecular basis to understand the regulatory mechanisms of endogenous formaldehyde generation in *L. edodes* during the growth process.

## 2. Results and Discussion

### 2.1. Gene Expression of Ggtl and Csl

**Figure 2:**
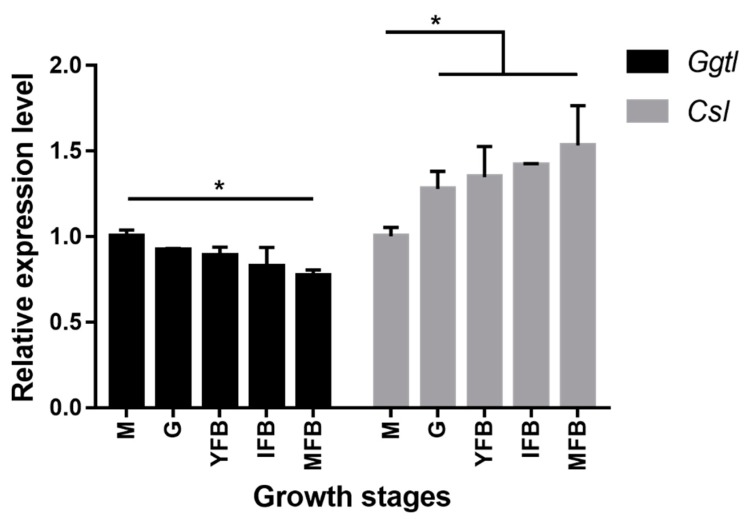
Expression of *Ggtl* and *Csl* during fruit-body development of *Lentinula edodes* strain W1. Relative expression during five growth stages, M, mycelia; G, grey; YFB, young fruiting body; IFB, immature fruiting body; MFB, mature fruiting body. Transcript levels of *Ggtl* (black bars) and *Csl* (gray bars) were determined by real-time quantitative RT-PCR analysis and normalized against *Actinl*. The expressions during the M stage were taken as 1. Error bars indicate standard deviation for three independent experiments. *p < 0.05, ANOVA tests by Duncan’s indicate significant differences.

The expressions of *Ggtl* (encoding Leggt) and *Csl* (encoding Lecsl) during fruiting body development were analyzed by examining their transcript levels using real-time quantitative PCR. Both *Ggtl* and *Csl* were expressed at all the five stages of fruit-body development: mycelia, grey, young fruiting body, immature fruiting body and mature fruiting body, but differed in their expression patterns (Figure 2). Specifically, the transcript level of *Ggtl* decreased during the growth process, in contrast to an increase for *Csl*. Additionally, *Ggtl* showed the highest and lowest expression level in mycelia and mature fruiting body, respectively, which was just the opposite for *Csl*. There was approximately 1.5-fold difference between the highest and lowest expression levels in the two genes. Analysis of variance showed a significant difference in the *Ggtl* and *Csl* expression levels between mycelia and fruiting body stages, while *Ggtl* exhibited a significantly different expression in the four fruiting body stages (*p* < 0.05).

Based on our in-house transcriptome data, the expression pattern of *Ggtl* in different growth stages was basically in line with the quantitative RT-PCR results of our experiment. *Csl* was first reported as a gene involved in the generation of unique aroma of *L. edodes* [19]. In an early report, *Csl* displayed no obvious change in the expression level at 1, 2 and 3 h in the stage of mycelium or fruiting body during hot-air drying [20], which were basically consistent with our research results. Overall, there was a gradual decline of *Ggtl* expression level in different growth stages and an obvious increase of *Csl* expression level between mycelia and fruiting body stages.

### 2.2. Western Blot of Leggt and Lecsl

**Figure 3:**
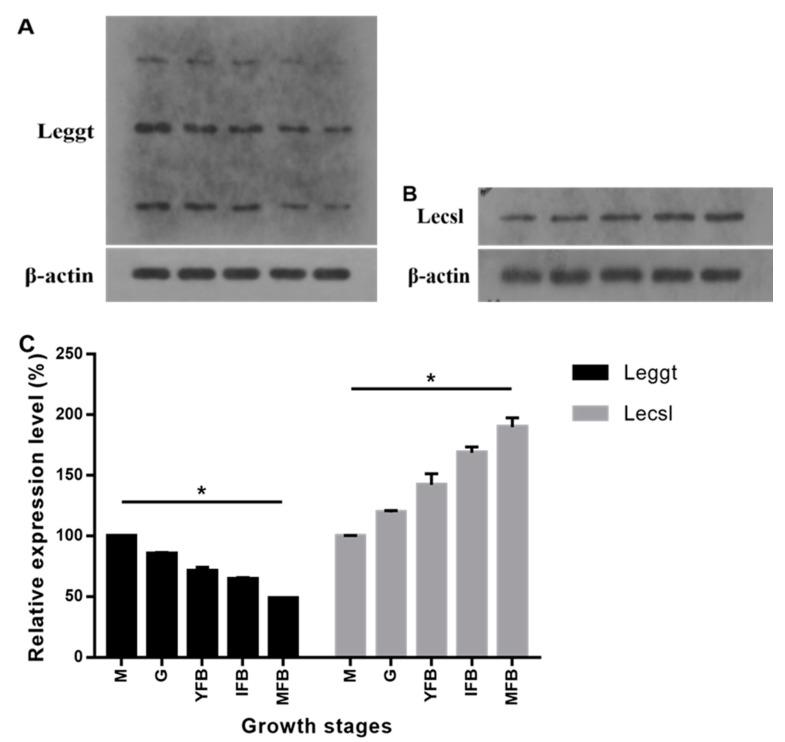
(**A**) Protein levels of Leggt at five stages of growth. (**B**) Protein levels of Lecsl at five stages of growth. (**C**) Relative expression of Leggt and Lecsl during five growth stages. β-actin protein was used as loading control, and the expressions during the M stage were taken as 100%. M, mycelia; G, grey; YFB, young fruiting body; IFB, immature fruiting body; MFB, mature fruiting body. Error bars indicate standard deviation for three independent experiments. *p < 0.05, ANOVA tests by Duncan’s indicate significant differences.

The protein expression levels of Leggt (GGT protein of *L. edodes*) and Lecsl (C-S lyase protein of *L. edodes*) in *L. edodes* at different growth stages were determined by Western blot using β-actin as an internal reference (Figure 3A,B) [21]. Notably, Leggt exhibited three bands, which conformed to previous reports [22,23], and their gray values showed a gradual decrease in the five stages. A previous study has shown that a mature gamma-glutamyl transpeptidase consists of one polypeptide chain and can be divided into a large and a small subunit by self-catalysis at the highly conserved threonine [24]. Correspondingly, three bands were shown in our Western blot analysis of Leggt. The protein expression levels showed a decrease in Leggt while a gradual increase in Lecsl during the growth process of *L. edodes* (Figure 3C). The overall trend of protein expression levels was similar to that of mRNA expression levels. Moreover, the two proteins showed significant differences in all the five samples. The changes between the highest and lowest expression levels of Leggt and Lecsl were 2.1-fold and 1.9-fold, respectively. This is the first report on the expression levels of these two enzyme proteins in shiitake mushrooms. Our data indicated that the expression of Leggt decreased while Lecsl increased across the five growth stages of *L. edodes*.

### 2.3. Enzyme Activities of GGT and C-S Lyase

**Figure 4:**
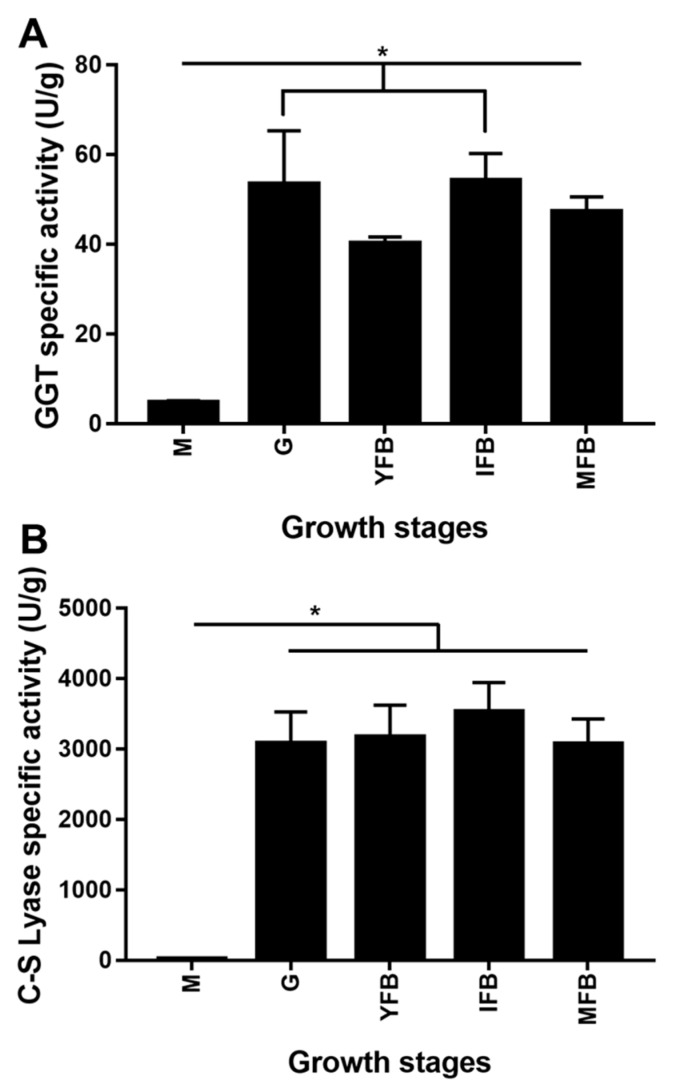
(**A**) The specific activity of GGT at the five growth stages. The reaction mixture containing the enzyme and the GPNA substrate was analyzed under standard conditions, and the residual activity was calculated. (**B**) The specific activity of C-S lyase at the five growth stages. The reaction mixture containing the enzyme and S-ethyl-L-cysteine sulfoxide substrate was analyzed under standard conditions, and the residual activity was calculated. M, mycelia; G, grey; YFB, young fruiting body; IFB, immature fruiting body; MFB, mature fruiting body. Error bars indicate standard deviation for three independent experiments. *p < 0.05, ANOVA tests by Duncan’s indicate significant differences.

Figure 4A,B show the activity of GGT and C-S lyase at five different growth stages of *L. edodes*. The enzyme activities of GGT and C-S lyase were the lowest at the mycelia stage (4.7 U/g of GGT, 17.1 U/g of C-S lyase) and showed an obvious increase at the four fruiting body stages. Moreover, the GGT enzyme activity was relatively lower at the young fruiting body stage, while the C-S lyase enzyme activity showed no significant differences at the later four fruiting body stages (*p* < 0.05).

Our experimental data (40.2–54.1 U/g) were consistent with the results of Huang et al., who reported that the GGT enzyme activity at the four fruiting body stages ranged from 40 to 80 U/g, with a similar difference at each stage in the fruiting body samples [25]. This is the first report about the C-S lyase enzyme activities of *L. edodes* at different growth stages. The C-S lyase enzyme activities in the present study (17.1–3575.6 U/g) were obviously lower than those determined by Xu et al. under high-temperature pre-drying (45, 55, 65 and 75 °C for 30 min) of air-dried (45 °C for 4.5 h, 60 °C for 4 h) *L. edodes* (80.17–100.54 U/mg) [26]. Liu et al. reported that the C-S lyase from *L. edodes* showed the optimum activity at 40 °C and was stable at 20–60 °C [18]. *Csl* has been demonstrated as a heat-inducible gene [27], so the activity of protein encoded by it could be improved greatly. In this study, our samples were generally collected at 25 °C, which was far below the drying treatment temperature (>45 °C), so the C-S lyase enzyme activity was lower than that treated under high temperature. Collectively, the C-S lyase enzyme activity showed no significant difference at the four fruiting body stages (*p* < 0.05).

However, there was a marked difference between the two enzymes activities and their expression levels at the mycelia stage. GGT performs different functions in peptide transferase reaction and hydrolysis reaction under different conditions [16]. Lecsl has already been demonstrated to have one active center involved in the binding of the two substrates, S-methyl-L-cysteine sulfoxide and L-cysteine, with both cysteine sulfoxide lyase and cysteine desulfurase activities [19]. In addition, there are many factors affecting enzyme activity, such as pH, temperature, metal ions and so on. The two enzymes activities were reported to be stimulated by Na^+^, K^+^, Mg^2+^ and Ca^2+^ ions [18]. The environment conditions are very different between the mycelia stage and the four fruiting body stages, since the former belongs to vegetative growth and the latter belong to reproductive growth. These observations suggested that there might be a sort of regulatory mechanism that activated the two enzymes during the fruiting body stages while stayed inactive at the mycelial stage, which we failed to detect in this study.

### 2.4. Endogenous Formaldehyde Content in L. edodes

**Figure 5:**
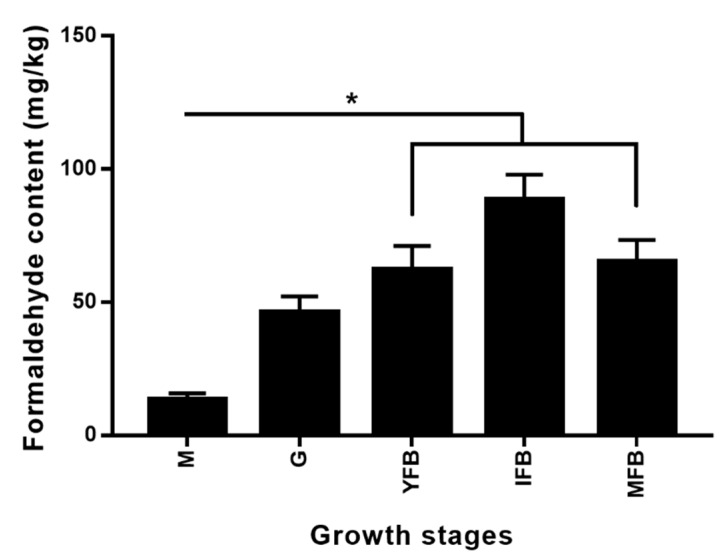
Endogenous formaldehyde content of *L. edodes* strain W1 at different growth stages. M, mycelia; G, grey; YFB, young fruiting body; IFB, immature fruiting body; MFB, mature fruiting body. Error bars indicate standard deviation for three independent experiments. *p < 0.05, ANOVA tests by Duncan’s indicate significant differences.

Figure 5 showed the content of endogenous formaldehyde in *L. edodes* at different growth phases. Compared with the mycelia stage, the endogenous formaldehyde content increased significantly (*p* < 0.05) at the four fruiting body stages, reached the maximum at the immature fruiting body stage and slightly decreased at the mature fruiting body stage. The trend of formaldehyde content at these four stages accorded with the findings of Huang et al. and Li et al. [25,28], which ranged from 13 to 89 mg/kg (dry weight) at all five stages. Mason et al. determined the formaldehyde content as 8–24 mg/kg in fresh shiitake mushrooms [29].

The endogenous formaldehyde content at different growth stages showed a similar change trend to that of C-S lyase enzyme activity. The formaldehyde content of *L. edodes* during the drying has been reported to range between 150 and 400 mg/kg (dry weight) [26]. The increase of the two enzyme activities in the drying process also led to a significant increase in the endogenous formaldehyde content. Xu et al. indicated that GGT and C-S lyase were involved in formaldehyde formation and their activities were positively correlated with formaldehyde content [26]. Although the activities of the GGT and C-S Lyase were higher in IFB than in the other four stages, the differences were not significant. The endogenous formaldehyde was found to be produced from oxidative decomposition of the folate backbone and creates a benign 1C unit that can sustain essential metabolism in human cells [30]. Additionally, *L. edodes* also contains folic acid. Therefore, whether there are other enzymes and metabolic pathways involved in the generation of endogenous formaldehyde in *L. edodes* needs to be further studied.

### 2.5. Correlation Analysis

The effects of GGT and C-S lyase on the generation of endogenous formaldehyde in *Lentinula edodes* at different growth stages were intuitively determined by correlation analysis (Table 1). The formaldehyde content of *L. edodes* showed a positive and significant (*p* < 0.01) correlation (R) with the expression level of *Csl* and Lecsl and the activity of C-S lyase and GGT (0.746, 0.805, 0.867 and 0.768, respectively), while a negative relationship with the expression level of *Ggtl* and Leggt (−0.699 and −0.787; *p* < 0.01).

Japanese researchers pointed out that the formaldehyde content of *L. edodes* was stable during the growth process. However, the formaldehyde content after the drying process showed 3–4-fold increase. For example, in the dried shiitake mushrooms, the formaldehyde content ranged from 100 to 230 mg/kg, in contrast to 8–24 mg/kg in fresh ones [14]. Xu et al. indicated that the enzyme activities of GGT and C-S lyase were much higher under high temperature (>45 °C) than under 25 °C. These results demonstrated that the activation of the two key enzymes promoted reactions, leading to the production of a large amount of formaldehyde in *L. edodes* [26], which was well supported by our results in this study. This is the first report to show that the mRNA and protein expression levels of C-S lyase had significant and positive effects on the endogenous formaldehyde content of mushrooms.

Although the mRNA and protein expression levels of GGT were shown to be negatively correlated with the formaldehyde content, both GGT and C-S lyase were proved to be indispensable for the generation of endogenous formaldehyde in *L. edodes.* As previously reported, only the joint action of the two enzymes could promote the generation of endogenous formaldehyde [18], and GGT was the rate-limiting enzyme in the synthesis process of endogenous formaldehyde in *L. edodes* [29]. Our results showed that the activities of both of GGT and C-S lyase played a positive role in endogenous formaldehyde generation, implying the crucial effects of GGT in this process. GGT was also reported to be implicated in the transfer of amino acids across the cellular membrane and in metabolism of glutathione to cysteine by cleaving the glutamyl amide bond to preserve intracellular homeostasis by oxidative stress [31,32]. Besides, the transcription and function of genes are not synchronized in time and space. The presence of *Ggtl* homologous genes was also reported [33]. Moreover, compared with C-S Lyase, GGT has a much more complex structure and function. Despite the negative correlation of *Ggtl* and Leggt expression levels, we could not neglect their effects on the endogenous formaldehyde content in the mushroom. For a better control on the generation of endogenous formaldehyde in *L. edodes,* further studies should focus on the expression regulation of *Ggtl* and *Csl* at the transcription level.

Our study did not involve the influence of other potential metabolic pathways on the generation of endogenous formaldehyde, and whether other enzymes are implicated in the flavor metabolism pathways also needs to be investigated in future studies.

## 3. Materials and Methods

### 3.1. Fungal Strain and Culture Conditions

**Figure 6:**
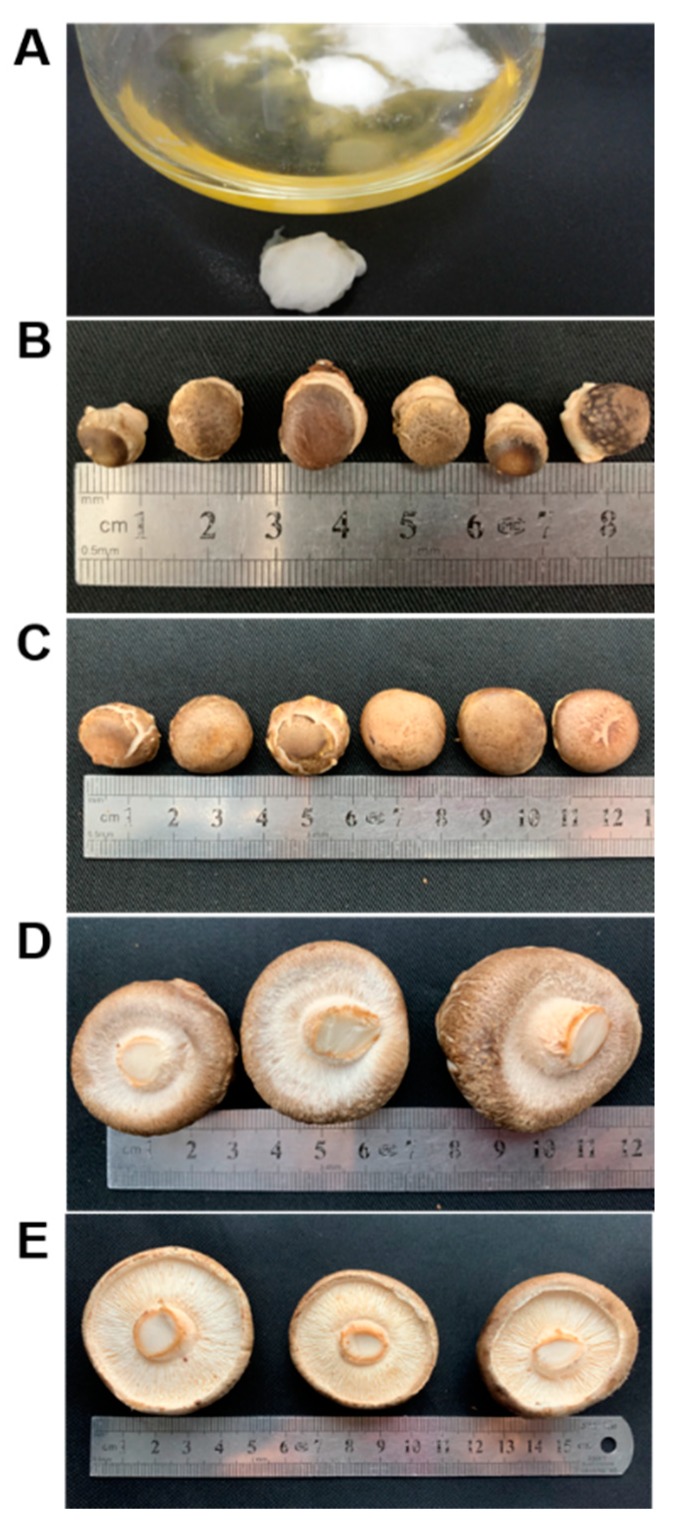
Five growth stages of *L. edodes* strain W1. (**A**) Mycelia. (**B**) Grey (5–10 mm in cap diameter). (**C**) Young fruiting body (15–20 mm in cap diameter). (**D**) Immature fruiting body (with partial veil not ruptured). (**E**) Mature fruiting body (with partial veil entirely ruptured).

A dikaryotic strain of basidiomycete *Lentinula edodes* strain W1 (preserved in the Institute of Applied Mycology, Huazhong Agricultural University, Wuhan, China) was used in this study [34]. The *L. edodes* samples were obtained at five different stages: mycelia (used as control) and four fruiting body stages (grey, young fruiting body, immature fruiting body and mature fruiting body). Briefly, the mycelia were cultivated on 25 mL CYM liquid medium (2% glucose, 0.2% yeast extracts, 0.2% peptone, 0.1% K_2_HPO_4_, 0.05% MgSO_4_ and 0.046% KH_2_PO_4_) in a conical flask and collected after growth of 12 days. Next, a conventional fruiting treatment was conducted as previously described [35]. The samples of grey (5–10 mm in cap diameter), young fruiting body (15–20 mm in cap diameter), immature fruiting body (with partial veil not ruptured) and mature fruiting body (with partial veil entirely ruptured) were harvested separately during fruiting treatment (Figure 6) [36]. The collected mushroom samples were immediately frozen in liquid nitrogen and stored at −80 °C for further use. All samples were collected in three biological replications.

### 3.2. RNA Isolation and Real-Time Quantitative PCR

Total RNA was isolated using RNAiso plus (TaKaRa, Kusatsu, Japan) according to the manufacturer’s instructions [37]. The total RNA concentration and purity were detected using a Nano Drop 2000 spectrophotometer (Thermo Scientific, Wilmington, DE, USA; 2.0 < A260/A280 < 2.2). The integrity of RNA was checked by electrophoresis on 1% agarose gel, and the three bands of 28S, 18S and 5S could be clearly observed (Supplementary Figure S1).

Then, 20 μL cDNA was synthesized from 1 μg of total RNA using the HiScript II Q RT SuperMix for qPCR (+ gDNA wiper) kit (Vazyme Biotech, Nanjing, China) according to the manufacturer’s instructions. Next, the cDNA was two-fold diluted with double-distilled water and stored at −20 °C for quantitative RT-PCR analysis. Specific primers were designed for quantitative RT-PCR analysis of the tested genes, such as *Ggtl,* encoding Leggt (γ-glutamyl transpeptidase); *Csl*, encoding Lecsl (*L. edodes* C-S lyase, *L. edodes* genome Gene ID: LE01Gene02830) and β-actin gene (*Actinl*, encoding *L. edodes* β-actin, *L. edodes* genome Gene ID: LE01Gene01050; Supplementary Table S1) [33].

Quantitative RT-PCR was performed using a CFX Connect real-time PCR system (BIO-RAD). Each reaction consisted of 0.4 μL each of the forward and reverse primers (10 μM), 1 μL of two-fold diluted cDNA, 5 μL of 2 × Taq Master Mix (Vazyme Biotech, Nanjing, China) and 3.2 μL of double-distilled water. The qRT-PCR was performed at 95 °C for 3 min, followed by 40 cycles of 95 °C for 20 s, 60 °C for 30 s, 72 °C for 30 s and then maintaining at 72 °C for 10 min in a 96-well reaction plate. The specificity and identity of PCR products were verified by melting curve analysis to distinguish specific PCR products from the primer dimmer-caused nonspecific PCR. The existence of a single peak proved each PCR product was specific.

The relative expression was calculated using the 2^−ΔΔCT^ method as previously described [38]. The expression of *Actinl* was used as an internal reference [39]. The expressions during the mycelium stage were taken as control. All PCR experiments were performed in three biological and three technical replications (the maximum difference in Ct was 0.5).

### 3.3. Extraction of Total Protein and Western Blot Analysis

Total protein of *L. edodes* was extracted as previously reported [40]. Briefly, 0.1 g of mycelia or fruiting body powder from each group (three replicates for each group) was mixed with 0.5 mL of extraction buffer (0.5 M Tris-HCl, 50 mM EDTA, 0.1 M NaCl and 40 mM dithiothreitol). The supernatants were collected after extraction for 10 min and centrifugation at 10,000× *g* for 15 min at 4 °C to remove the insoluble substance. Next, the same volume of saturated Tris-phenol was added to the supernatants, followed by the addition of five volumes of pre-cooled 0.1 M ammonium acetate in methanol to precipitate the protein. After washing with pre-cooled 80% acetone several times, the precipitated proteins were resolubilized and denatured for 10 min in 40 µL solution buffer (7 M urea, 50 mM Tris-HCl, 25 mM EDTA, 10 mM NaCl and 60 mM dithiothreitol). Finally, the pelleted proteins were diluted to 200 µL for further analysis. The concentration of the total protein was tested by the Coomassie Brilliant Blue G250 method [41], and the quality of protein was checked by 10% SDS-PAGE (Supplementary Figure S2) [42].

Western blot was used to analyze the expression of γ-glutamyl transpeptidase (Leggt, EC 2.3.2.2) and S-alkyl-L-cysteine sulfoxide lyase (Lecsl, EC 4.4.1.4) at different growth stages of *L. edodes.* After 50 μg of each protein sample was run on 10% SDS-PAGE gels (Bio-Rad Mini, Hercules, CA, USA), Western blot was performed by standard protocols using 1:200 anti-Leggt and anti-Lecsl polyclonal antibody sera. The antibodies against Leggt and Lecsl were raised by immunizing rabbits with the mixture of purified recombinant protein, which was expressed in *Escherichia coli* BL21 and purified by Ni-NTA Agarose column (Genscript, Nanjing, China) and Freund’s adjuvant [43]. The specificity of polyclonal antibodies was detected by Western blot. The results showed that anti-Leggt polyclonal antibody sera had special bands at 68 kDa, 45 kDa and 23 kDa and anti-Lecsl polyclonal antibody sera had special band at 54 kDa, respectively. 1:50,000 horseradish peroxidase conjugated secondary antibody (BOSTER, Wuhan, China). Meanwhile, the β-actin antibody (BOSTER, Wuhan, China) was treated with the same protocol as an internal control [21].

### 3.4. Enzyme Activity Assays

GGT activity was determined by the transfer rate of γ-glutamyl from γ-glutamyl *p*-nitroanilide (GPNA) as reported by Liu et al. [18]. The mixture including 1 mL crude enzyme extract from *L. edodes*, 1 mL GPNA (3.5 mM) and 3 mL Tris-HCl (0.5 M, pH = 7.6) was incubated at 37 °C for 20 min and the reaction was stopped by adding 3 mL of 1.5 M cold (4 °C) acetic acid. Then, the amount of p-nitroaniline released was measured at 410 nm. The specific activity of GGT was defined as the amount of enzyme that released 1 μmol of p-nitroaniline from the substrate per min per g protein (U/g).

C-S lyase activity was measured as previously described with some modifications [44]. The mixture containing 0.3 mL crude enzyme extract from *L. edodes*, 0.5 mL S-ethyl-L-cysteine sulfoxide and 0.2 mL Tris-HCl (0.5 M, pH = 7.6) was incubated at 37 °C for 5 min. The reaction was terminated by adding 1 mL trichloroacetic acid (TCA, 10%). After supplementation with 1 mL 2,4-dinitrophenylhydrazine (DNPH, 0.1%, *m*/*v*) were added to the mixture was incubated for 5 min at 25 °C. Finally, 2.5 mL NaOH (2.5 M) was added to the mixture and incubated for 10 min at 25 °C. The absorbance of DNPH at 520 nm was measured. The specific activity of C-S lyase was expressed as units of enzyme per g of *L. edodes* protein (U/g).

### 3.5. Determination of Endogenous Formaldehyde Content in L. edodes

Steam distillation was used to extract formaldehyde from *L. edodes* at each growth stage. Each sample was supernatant of 4 g *L. edodes* homogenized with 100 mL Tris-HCl (0.5 M, pH = 7.6) buffer and 10 mL 10% (*v*/*v*) phosphoric acid aqueous solution in a 250 mL distillation flask. Water vapor was collected into a 150 mL flask, and then immersed in an ice-bath. The distillation process was stopped when 6070 mL of the distillate was collected and made up to 100 mL by deionized water. Formaldehyde in the distillate was derived by adding 1 mL of the distillate, 3.5 mL acetate buffer (0.1 M, pH = 4.0) and 0.5 mL DNPH (3 mg/mL) into a centrifuge tube at 25 °C for 15 min. Then the derived sample was filtered through a 0.22 μm filter for HPLC analysis. The formaldehyde derivative (formaldehyde-DNPH) of each group was separated and determined by a reverse-phase HPLC system (Waters, Milford, MA, USA). The mobile phase was composed of 0.05% acetic acid in acetonitrile and 0.05% acetic acid in water. The injection volume was 20 μL. All samples were detected at 355 nm as previously reported [18].

### 3.6. Data Analysis

All experimental data were presented as the mean ± standard deviation from at least three independent experiments. The ANOVA tests of statistical significance were performed by Duncan’s multiple range tests using SPSS 20.0. *p*-values of <0.05 and <0.01 were accepted as significant and remarkable significant difference, respectively. The correlations of formaldehyde content with the expression levels of *Ggtl*, *Csl*, Leggt and Lecsl as well as GGT and C-S lyase activities were analyzed separately by Pearson correlation coefficient and trend of data using SPSS 20.0.

## 4. Conclusions

In this study, we reported for the first time the mRNA and protein expression levels and the activities of GGT and C-S lyase as well as their correlations with the endogenous formaldehyde content in *L. edodes* at different growth stages. The protein expression levels of Leggt and Lecsl were consistent with the mRNA expression levels of *Ggtl* and *Csl*. Additionally, the expression levels of GGT were decreased while those of C-S lyase were increased with the growth and development of *Lentinula edodes*. Furthermore, the enzyme activities and formaldehyde content were found to be the lowest in the mycelium stage. Our results demonstrated that the expression levels of *Csl* and Lecsl as well as the enzyme activities of C-S lyase and GGT were positively correlated with formaldehyde content during the development of *L. edodes*. These findings revealed the role of GGT and C-S lyase in generating endogenous formaldehyde at the molecular level. They also provided a molecular basis for regulating endogenous formaldehyde in the process of *L. edodes* growth.

## Figures and Tables

**Table 1:** Correlations (R) of the formaldehyde contents in *L. edodes* with *Ggtl* expression levels, *Csl* expression levels, Leggt expression levels, Lecsl expression levels, GGT enzyme activities and C-S lyase enzyme activities at different growth stages.

Properties	Formaldehyde Content
*Ggtl* expression levels (mRNA)	−0.699 **
*Csl* expression levels (mRNA)	0.746 **
Leggt expression levels (Protein)	−0.787 **
Lecsl expression levels (Protein)	0.805 **
GGT enzyme activities	0.768 **
C-S lyase enzyme activities	0.867 **

** significant at 0.01 level.

## Supplementary Materials

The supplementary materials are available online. Supplementary Figure S1: Agarose gel electrophoresis of RNA extracted from *L. edodes* at different growth stages; Supplementary Figure S2: SDS-PAGE of *L. edodes* total protein at different growth stages; Supplementary Table S1: Quantitative primer information of *Ggtl, Csl* and *Actinl*.
