# Supplementary material for: Effects of GGT and C-S Lyase on the Generation of Endogenous Formaldehyde in Lentinula edodes at Different Growth Stages
Source: Molecules. 2019 Nov 20;24(23):4203. doi: 10.3390/molecules24234203 (PMC6930676; doi:10.3390/molecules24234203)
Supplement: Supplementary file 1 [file molecules-24-04203-s001.pdf]

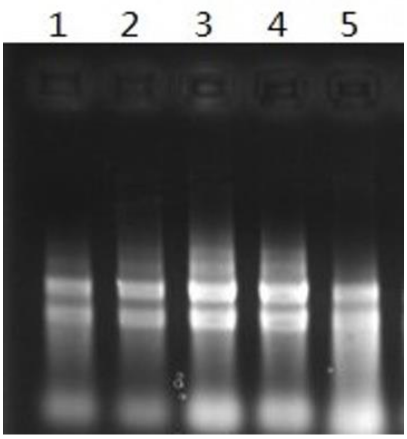

Supplementary Figure S1. Agarose gel electrophoresis of RNA extracted from *L. edodes* at different growth stages. Lane 1, total RNA of mycelia; lane 2, total RNA of grey; lane 3, total RNA of young fruitbody; lane 4, total RNA of immature fruitbody; lane 5, total RNA of mature fruitbody.

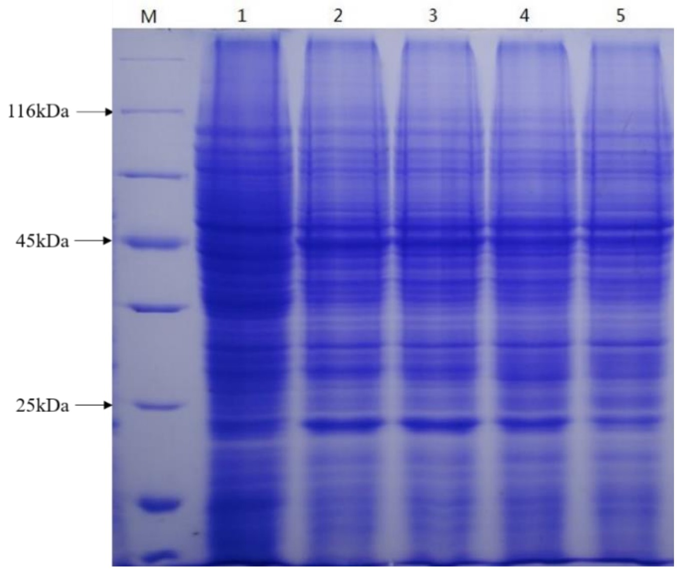

Supplementary Figure S2. SDS-PAGE of *L. edodes* total protein at different growth stages. Lane M, protein markers; lane 1, total protein of mycelia; lane 2, total protein of grey; lane 3, total protein of young fruitbody; lane 4, total protein of immature fruitbody; lane 5, total protein of mature fruitbody.

Supplementary Table S1. Quantitative primer information of *Ggtl, Csl* and *Actinl*

Primer name	Primer sequences (5' to 3')
Ggtl-qRT-Forward	TTAGCGACTGAGGCTGGAC
Ggtl-qRT-Reverse	CCTCCAATGCCACAACAA
Csl-qRT-Forward	AATGGGTCGGCGGAGAAG
Csl-qRT-Reverse	TGCCGTGCCAACTGAAGC
Actinl-qRT-Forward	GGAGAAGATTTGGCATCACACA
Actinl-qRT-Reverse	GAAGAGCGAAACCCTCGTAGA
